# Mixed 20-peptide cancer vaccine in combination with docetaxel and dexamethasone for castration-resistant prostate cancer: a randomized phase II trial

**DOI:** 10.1007/s00262-020-02498-8

**Published:** 2020-02-05

**Authors:** Masanori Noguchi, Gaku Arai, Shin Egawa, Chikara Ohyama, Seiji Naito, Kazumasa Matsumoto, Hirotsugu Uemura, Masayuki Nakagawa, Yasutomo Nasu, Masatoshi Eto, Shigetaka Suekane, Tetsuro Sasada, Shigeki Shichijo, Akira Yamada, Tatsuyuki Kakuma, Kyogo Itoh

**Affiliations:** 1 Canver Vaccine Center, Kurume University School of Medicine, 67 Asahi-machi, Kurume, 830-0011 Japan; 2 Department of Urology, Kurume University School of Medicine, 67 Asahi-machi, Kurume, 830-0011 Japan; 3 Cancer Vaccines, Research Center for Innovative Cancer Therapy, Kurume University School of Medicine, Kurume, Japan; 4 Biostatistics Center, Kurume University School of Medicine, Kurume, Japan; 5 Department of Urology, Dokkyo Medical University Koshigaya Hospital, Koshigaya, Japan; 6 Department of Urology, Jikei University School of Medicine, Tokyo, Japan; 7 Department of Urology, Graduate School of Medicine and School of Medicine, Hirosaki University, Hirosaki, Japan; 8 Sanshinkai Hara Hospital, Fukuoka, Japan; 9 Department of Urology, Kitasato University School of Medicine, Kanagawa, Japan; 10 Department of Urology, Kinki University Faculty of Medicine, Osaka, Japan; 11 Department of Urology, Kagoshima University Graduate School of Medical and Dental Science, Kagoshima, Japan; 12 Department of Urology, Okayama University Graduate School of Medicine, Okayama, Japan; 13 Department of Urology, Graduate School of Medical Sciences, University of Kyushu, Fukuoka, Japan; 14 Kanagawa Cancer Center Research Institute, Kanagawa, Japan

**Keywords:** Prostate cancer, Multiple-peptide vaccine, Immunotherapy, Docetaxel, Phase II trial

## Abstract

A novel cancer vaccine consisting of 20 mixed peptides (KRM-20) was designed to induce cytotoxic T lymphocytes (CTL) against twelve different tumor-associated antigens. The aim of this phase II trial was to examine whether KRM-20 in combination with docetaxel and dexamethasone enhances the antitumor effects in patients with castration-resistant prostate cancer (CRPC). In this double-blind, placebo-controlled, randomized phase II study, we enrolled chemotherapy-naïve patients with CRPC from ten medical centers in Japan. Eligible patients were randomly assigned 1:1 centrally to receive either KRM-20 combined with docetaxel and dexamethasone (*n* = 25) or placebo with docetaxel and dexamethasone (*n* = 26). The primary endpoint was the difference in prostate-specific antigen (PSA) decline between each treatment. The rates of > 50% PSA decline in the two arms were similar (56.5% versus 53.8%; *P* = 0.851). Human leukocyte antigen (HLA)-matched peptide-specific immunoglobulin G (*P* = 0.018) and CTL (*P* = 0.007) responses in the KRM-20 arm significantly increased after treatment. The addition of KRM-20 did not increase toxicity. There were no between-group differences in progression-free or overall survival (OS). The addition of KRM-20 was safe, and similar PSA decline and HLA-matched peptide-specific CTL and IgG responses increased in combination with docetaxel and dexamethasone in CRPC patients. Subgroup analysis suggested that this treatment is favorable for CRPC patients with ≥ 26% lymphocytes or PSA levels of < 11.2 ng/ml, but further clinical trials comparing OS are required.

**Electronic supplementary material:**

The online version of this article (10.1007/s00262-020-02498-8) contains supplementary material, which is available to authorized users.

## Introduction

Prostate cancer-related death is common among patients with metastatic castration-resistant prostate cancer (CRPC) in which the disease progresses despite androgen deprivation therapy. Although several new agents for CRPC, such as sipuleucel-T, cabazitaxel, abiraterone acetate, enzalutamide, and radium-223, have been approved recently based on their demonstrated overall survival (OS) benefits in phase 3 studies, each of these prolonged survival by only a few months [2–6]. Thus, there remains a need for treatments that can provide stable disease control and long-term survival benefits. After the approval of sipuleucel-T for patients with asymptomatic or minimally symptomatic CRPC based on survival benefits [2], and stable responses observed with the checkpoint inhibitors ipilimumab and nivolumub in other malignancies [7–9], immunotherapy has emerged as a viable and attractive strategy for the treatment of CRPC. However, immunotherapy alone may be unable to induce an immune response of sufficient potency to result in tumor regression when immune tolerance and large tumor burden are present.

The standard first-line chemotherapy for patients with progressive CRPC has consisted of docetaxel and oral prednisone [10, 11]. Several animal studies have examined the use of cancer vaccines in combination with docetaxel, and reported enhancement of T-cell responses with antitumor activity [12–14]. These studies suggested that docetaxel is able to reduce the tumor burden and immune-suppressing elements, such as the increase in myeloid-derived suppressor cells (MDSC) in the tumor microenvironment, and it is well known that tumor-associated immunosuppression plays a significant role in tumor progression and resistance to immunotherapy [13].

We developed a novel cancer vaccine consisting of 20 mixed peptides (KRM-20) for patients with CRPC designed to induce cytotoxic T lymphocytes (CTL) against twelve different tumor-associated antigens (TAAs) highly expressed in prostate cancer tissues. The CTL epitopes represented by these 20 peptides are restricted to human leukocyte antigen (HLA)-A2, -A24, -A3 super type (-A3, -A11, -A31 or -A33) or -A26 of major histocompatibility complex class 1 molecules, providing coverage of the majority of patients who have different HLA alleles. A previous phase I study on KRM-20 for patients with CRPC demonstrated the feasibility, safety, and rapid and high immune responses without changes in immunosuppressive cell subsets [15].

This phase II study was designed to evaluate if docetaxel has the ability to alter components of the immune system independent of antitumor activity, and to examine the potential synergistic activity of the 20-mixed peptides vaccine in combination with docetaxel and dexamethasone.

## Patients and methods

### Patient population

For this phase II, randomized, double-blind, placebo-controlled study, we enrolled chemotherapy-naïve patients with CRPC from ten medical centers in Japan. The subjects must satisfy the following conditions: (1) patients must be diagnosed as prostate cancer pathologically at the initial treatment; (2) patients who had progressive disease after androgen deprivation therapy (ADT) either by surgical castration, gonadotropin-releasing hormone or antagonist treatment. Progressive disease while receiving ADT, defined by any 1 of the following: (1) at least two consecutive rises in serum PSA obtained at a minimum of 1-week intervals; (2) measurable disease with ≥ 50% increase in the sum of the cross products of all measurable lesions, or the development of new measurable lesions by RESIST; (3) non-measurable (bone) disease consisting of new areas of uptake by bone scan consistent with metastatic disease compared to previous imaging; (4) patients have serum PSA level ≥ 2 ng/m; (5) anti-androgen therapy is discontinued for at least 4 weeks before the first vaccination for patients receiving flutamide and 6 weeks for those receiving bicalutamide; (6) patients continue to stay on medical treatment such as LHRH agonists or LHRH antagonists to maintain testosterone level of 0.5 ng/m; (7) patients must be positive for HLA-A2, HLA-A24, HLA-A26 or HLA-A3 super type (A3, A11, A31, A33); (8) written informed consent must be obtained from patients; (9) patients must be more 20 year-old; (10) patients must be at a score level of 0 or 1 of an Eastern Cooperative Oncology Group (ECOG) performance status; (11) patients must be expected to survive more than 6 months; (12) patients must satisfy bone marrow function (white blood cell count ≥ 2500/mm^3^, lymphocyte count ≥ 1000/mm^3^, hemoglobin ≥ 8 g/dl, and platelets ≥ 100,000/mm^3^), hepatic function [total bilirubin ≤ 1.5 × the upper limit of normal (ULM), transaminase ≤ 2 × ULM], and renal function (serum creatinine ≤ 2 × ULM). Patients without previous bilateral orchiectomy continued receiving luteinizing hormone-releasing agonists. Exclusion criteria included acute infection, history of severe allergic reactions, pulmonary, cardiac or other systemic diseases, or other inappropriate conditions for enrollment as judged by clinicians.

### Study design and treatment

Randomization was performed centrally at the clinical research unit of Kurume University in Kurume, Japan. Patients were randomly assigned in a 1:1 ratio to receive either KRM-20 (study arm) or placebo (control arm) followed by intravenous docetaxel using a minimization technique with the following stratification factors: age (< 65 or ≥ 65 years old) and PSA (< 20 or ≥ 20 ng/ml). This study was double-blinded, and all physicians, patients, and investigators giving the interventions, assessing outcomes, and analyzing data were blinded to treatment assignment. In the event of a medical emergency in an individual patient, the treating physician was informed of the assigned treatment by a judge from the safety committee.

KRM-20 was designed to induce CTL against 20 peptides originating from twelve different TAAs, including PSA, prostatic acid phosphatase (PAP), prostate-specific membrane antigen (PSMA), epidermal growth factor-receptor (EGF-R), parathyroid hormone-related peptide (PTHrP), squamous cell carcinoma antigens 3 (SART3), cyclophilin B (CypB), Wolf-Hirshhorn syndrome critical region 2 (WHSC2), ubiquitin-conjugated enzyme variant Kua (UBE2V), heterogeneous nuclear ribonucleoprotein L (HNRPL), p56^*lck*^, and multidrug resistance-associated protein 3 (MRP3), as reported previously [15]. The name, source TAA, position, amino acid sequence, and HLA type of the KRM-20 including the 20 peptides are shown in Supplementary Table 1.

Patients received either KRM-20 (20 mg/0.5 ml) or placebo (0.5 ml) mixed with incomplete Freund's adjuvant (Montanide ISA-51VG; Seppic, Paris, France) subcutaneously on days 1, 8, 15, 22, and 29 with oral dexamethasone (1 mg) once daily on days 1–36. On day 36, one hour after intravenous docetaxel at 70 mg/m^2^, patients received subcutaneous KRM-20 or placebo injection. Treatment with docetaxel and the study drug was repeated every 3 weeks for up to five cycles, and patients continued oral dexamethasone once daily until the end of the study. Dosing delay and reduction for docetaxel was permitted if toxic effects were noted. It was possible to hold docetaxel for less than 2 weeks until recovery, or reduce it to 60 or 50 mg/m^2^ in the event of neutropenia (< 2000/mm^3^), platelets < 100,000/mm^3^, hemoglobin < 8 g/dl, total bilirubin > 1·5 × ULM, transaminase > 2 × ULM, or serum creatinine > 2 × ULM. If docetaxel was held for more than 3 weeks, the patient was removed from protocol treatment. Patients who received protocol treatment were followed-up for 3 years for survival analyses.

### Outcomes

For the primary endpoint of PSA decline, patients were evaluated at pre-treatment and every docetaxel cycle using serum PSA concentrations (at pre-treatment, and sixth to tenth and 3 weeks after protocol treatment). The rate of > 50% PSA decline was compared between arms. Patients were evaluated at pre-treatment and 3 weeks after protocol treatment by bone scans and computed tomography (CT) scans of the abdomen and pelvis followed by a 6-month interval. The secondary endpoints included immune responses, safety profile, progression-free survival (PFS), and OS. To assess immune responses during the protocol treatment, peripheral blood was collected at pre-treatment, and sixth, eighth, and tenth study drug injections. HLA-matched peptide-specific immunoglobulin G (IgG) titer in the plasma were measured using a Luminex system [16], and HLA-matched peptide-specific CTL in peripheral blood mononuclear cells (PBMC) was evaluated by IFN-γ ELISPOT assay, as described previously [15]. When the total HLA-matched peptide-specific IgG titers at the tenth study drug injection was higher than that at pre-vaccination, it was considered to be a positive response. Positive CTL responses were defined as a greater than 100-spot increase in the total number of HLA-matched peptide-specific IFN-γ spots at the tenth study drug injection. We also measured Treg and MDSC at the same points for exploratory analysis of immune suppression. Treg were defined as CD4+ CD25+ FoxP3+ cells among lymphocytes, and MDSC were identified as CD33+ 11b+ cells from the lineage markers (CD3, CD19, CD56, and CD16)- and HLA-DR-cells measured by multicolor flow cytometry. The safety profile was assessed throughout the study by monitoring for adverse events (AEs) [according to the National Cancer Institute Common Terminology Criteria for Adverse Events version 4.0 (NCI-CTC Ver. 4)], chemical laboratory tests, vital signs, and physical examinations. PFS was defined as the time in months from randomization until objective disease progression based on the PSA Working Group Consensus Criteria 2 (PCWG2) [17], the Response Evaluation Criteria in Solid Tumors (RECIST) 1·1 criteria, or death. OS was calculated as the time in months from the date of randomization to death or to the date of last contact for censored observations. Analyses of primary and secondary efficacy endpoints were based on the intention-to-treat (ITT) population that included all randomly assigned patients.

### Statistical design and analysis

The primary endpoint of this study was the comparison of each treatment arm for the rate of > 50% PSA decline from baseline. Based on the previous report [18], the assumed rate of > 50% PSA decline was 65% in the KRM-20 arm and 25% in the placebo arm. The target sample size was 50 assuming an ineligibility rate of approximately 10%. Sample size computation based on the large sample test was performed with the following assumptions: type I error rate = 0.05, power 80% and the ratio of the two groups as 1:1. The Student’s *t* test and chi-square test were used to compare quantitative and categorical variables among safety profiles and immune responses to the treatment, respectively. PFS and OS data for each arm were analyzed using the Kaplan–Meier method. The log-rank test was used for the comparison of the survival curves, and Cox proportional hazard analysis was used to estimate hazard ratios (HR). The confidence intervals (CI) reported were 95%. Cox proportional hazards regression model was used for univariate and multivariate analyses to identify factors that significantly impacted survival. All baseline parameters in the survival and proportional hazards regression analysis were analyzed as dichotomous variables using median or cut-off values. Statistical analyses were performed using SAS software version 9.1 (SAS Institute, Cary, NC, USA) with a two-sided significance level of 5%.

## Results

**Fig. 1:**
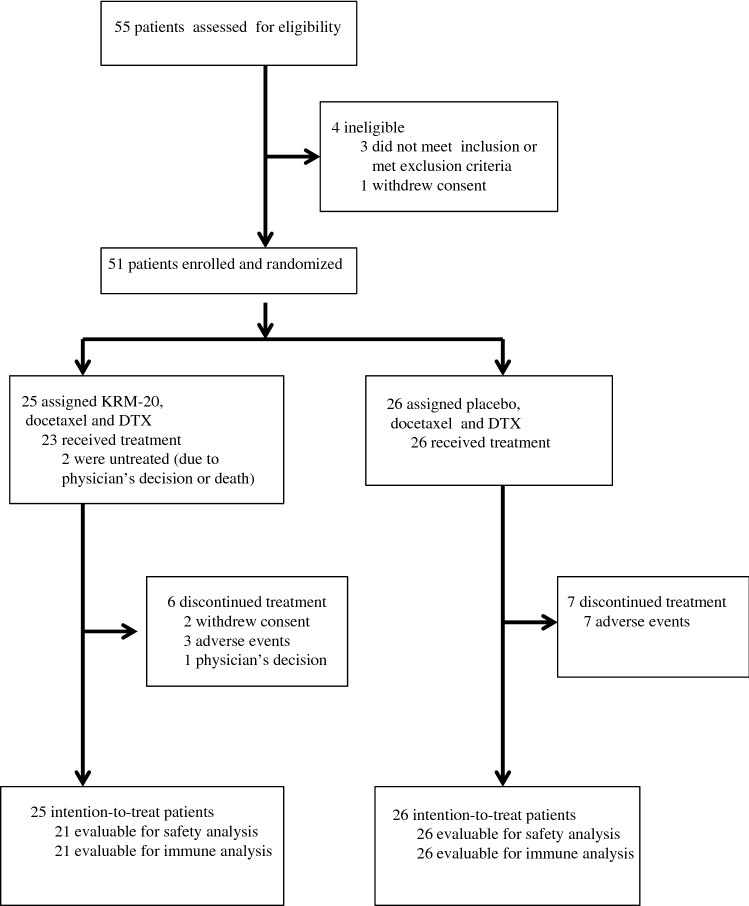
Study flowchart. *DTX* dexamethasone

Between July 31, 2013 and July 11, 2014, 55 chemotherapy-naïve patients with progressive CRPC were screened for enrollment at ten medical centers in Japan (Fig. 1). Fifty-one patients were enrolled and randomly assigned to receive either KRM-20 with docetaxel and dexamethasone (*n* = 25) or placebo (*n* = 26) with docetaxel and dexamethasone. Two patients in the KRM-20 arm were not treated due to the physician’s decision or death, and not included in the safety analysis set. Baseline demographic and clinical characteristics of participants, including median age, ECOG performance status, metastatic site, HLA types, Gleason scores, median PSA levels, median times from diagnosis to study entry, median IgG levels, and median CTL levels were balanced between the two arms (Table 1). Most patients (72% in the KRM-20 arm and 69.2% in the placebo arm) had bone disease, and all patients were refractory to previous hormone therapies. At the end of study treatment, 17 (73.9%) of 23 patients in the KRM-20 arm and 20 (76.9%) of 26 patients in the placebo arm had completed the study treatment (Fig. 1).

**Table 1:** Patient demographics and baseline characteristics

	KRM-20 arm (*n* = 25)	Placebo arm (*n* = 26)	Total (*n* = 51)
Age (years)			
Median	72	70	71
Range	41–85	51–81	41–85
ECOG performance status			
0	22 (88%)	23 (88.5%)	45 (88%)
1	3 (12%)	3 (11.5%)	6 (12%)
Time from diagnosis to study entry (years)			
Median	2	4	4
Range	0.6–12	0.8–9	0.6–12
Gleason score at diagnosis			
< 8	4 (16%)	5 (19%)	9 (18%)
≥ 8	19 (76%)	21 (81%)	40 (78%)
Unknown	2 (8%)	0	2 (4%)
HLA type			
A24	18 (72%)	17 (65.4%)	35 (67%)
A2	10 (40%)	10 (38.5%)	20 (39%)
A26	6 (24%)	5 (19.2%)	11 (22%)
A3 family	13 (52%)	13 (50%)	26 (51%)
Metastatic sites			
None	1 (4%)	4 (15.4%)	5 (10%)
Bone	18 (72%)	18 (69.2%)	36 (71%)
Lymph node	12 (48%)	12 (46.2%)	24 (47%)
Lung	2 (8%)	0	2 (4%)
Liver	1 (4%)	0	1 (2%)
Others	11 (44%)	8 (30.8%)	19 (37%)
Unknown	2 (8%)	0	2 (4%)
Previous treatment			
Hormone therapy	25 (100%)	26 (100%)	51 (100%)
Prostatectomy	4 (16%)	3 (11.5%)	7 (14%)
Radiation	9 (36%)	6 (23.1%)	15 (29%)
PSA (ng/ml)			
Median	11.2	10.1	11.2
Range	3.7–663.3	2.4–299.9	2.4–663.3
Neutrophils, %			
Median	63.8	67.4	65.0
Range	49.8–89	42.5–82.2	42.5–89
Lymphocytes, %			
Median	27.5	24.9	26.0
Range	6.3–42	13.3–49.4	6.3–49.4
IgG, FIU			
Median	1053	1282.5	1142
Range	519–57,231	136–92,009	136–92,009
CTL, spots			
Median	13	1	4
Range	0–280	0–53	0–280
MDSC, %			
Median	9.6	9.4	9.6
Range	1.3–22	0.1–20.8	0.1–22
Treg, %			
Median	2.7	3.2	2.8
Range	0.9–9.4	0.8–6.2	0.8–9.4

CTL, cytotoxic T lymphocytes; ECOG, Eastern Cooperative Oncology Group; FIU, fluorescence intensity units; HLA, human leukocyte antigen; IgG, immunoglobulin G; MDSC, myeloid-derived suppressor cells; Treg, regulatory T cells

The rates of > 50% PSA decline in the two arms were similar (56.5% vs. 53.8%), with no significant difference (*P* = 0.851, chi-square test).

**Fig. 2:**
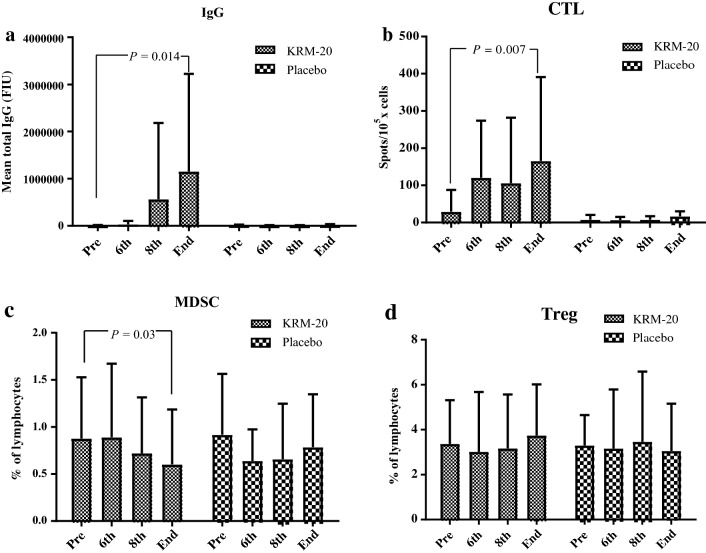
Immune responses in patients during treatment. **a** IgG responses in the KRM-20 arm significantly increased after treatment (*P* = 0.014, *t* test). **b** CTL responses in the KRM-20 arm significantly increased after treatment (*P* = 0.007, *t* test). **c** The number of MDSC in the KRM-20 arm significantly decreased after treatment (*P* = 0.03, *t* test). **d** The number of Treg in PBMC in both arms did not increase during treatment. *CTL* cytotoxic T lymphocytes, *IgG* immunoglobulin G, *MDSC* myeloid-derived suppressor cells, *PSA* prostate-specific antigen, *Treg* regulatory T cells

Regarding immune responses, the mean total HLA-matched peptide-specific IgG (*P* = 0.014, *t* test) and CTL (*P* = 0.007, *t* test) responses in the KRM-20 arm significantly increased after treatment, whereas the IgG and CTL responses in the placebo arm did not increase after treatment (Fig. 2a, b). The median number of HLA-matched peptides in the KRM-20 arm was 16 (8–17), and peptide-specific IgG and CTL responses matching HLA were observed in 8 (35%) of 23 patients and 5 (22%) of 23 patients, respectively (Supplementary Table 2). In the exploratory analysis for immune suppression, the numbers of both Treg and MDSC among PBMC in the two arms did not increase during treatment (Fig. 2c, d), and the number of MDSC in the KRM-20 arm significantly decreased after the treatment (*P* = 0.03, *t* test) (Fig. 2d).

AEs in the two arms during treatment are summarized in Supplementary Table 3. The most common AEs (occurring in more than 40% patients in one or both arms) were injection site reactions, alopecia, neutropenia, and peripheral neuropathy. AEs of grade 3 or higher developed in similar frequencies between the two arms: 16 (70%) of 23 patients in the KRM-20 arm and 18 (79%) of 26 patients in the placebo arm. Grade 5 events during treatment were observed in 2 patients with pneumonia in the placebo arm. The addition of KRM-20 did not increase toxicity. The dose of docetaxel was reduced due to hematological toxicity in a similar proportion in each patient arm (17.5% in the KRM-20 arm and 15% in the placebo arm).

**Fig. 3:**
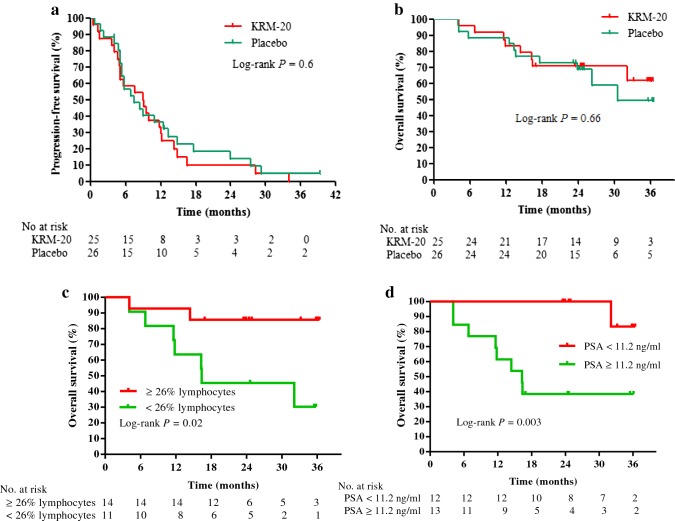
Kaplan–Meier curves. Progression-free survival (**a**) and overall survival (**b**) in the intention-to-treat population. Overall survival in KRM-20 patients according to cut-offs of 26% lymphocytes (**c**) or PSA level of 11.2 ng/ml (**d**). *PSA* prostate-specific antigen

After the median follow-up of 8.3 months (IQR 5.0–13.3), 45 (88%) of 51 patients had disease progression or died: 22 (88%) in the KRM-20 and 23 (85%) in the placebo arm. Based on investigator assessment of disease response and progression using PCWG2 or RECIST criteria, partial response was observed in 2 (8%) patients in the KRM-20 arm and 3 (12%) patients in the placebo arm. No complete responses were observed in either arm. The median PFS time was 8.9 months (95% CI 4.9–12.2) in the KRM-20 arm and 7.4 months (95% CI 5.3–12.5) in the placebo arm (Fig. 3a), but this difference was not significant (HR 1.0; 95% CI 0.6–1.9; *P* = 0.87). At the data cut-off date of July 1, 2017 after a median follow-up of 32.4 months (IQR 16.3–36.1), 24 (47%) patients in the ITT population had died: 12 (48%) patients in the KRM-20 arm and 12 (46%) patients in the placebo arm. The median OS time in the placebo arm was 30.5 months (95% CI 23.9–38.8), but it has yet to be reached in the KRM-20 arm (95% CI 16.4-not reached); it was estimated to be 37.7 months using the adjusted HR (Fig. 3b). Although patients in the KRM-20 arm had a slightly longer OS than those in the placebo arm, there was no between-group difference in median OS time (HR 0.81; 95% CI 0.3–2.1; *P* = 0.83). To further investigate the effects of KRM-20 with docetaxel and dexamethasone, Cox proportional hazards regression analysis was performed to find factors that can predict disease response in the KRM-20 arm. Univariate Cox analysis demonstrated % lymphocytes (*P* = 0.006) and PSA level (*P* = 0.003) to be significantly associated with survival. The factors with an HR less than 0.5 in the univariate analysis were included in the multivariate analysis of the model. In the KRM-20, ≥ 26% lymphocytes (HR 0.3; 95% CI 0.08–0.93; *P* = 0.04) and PSA levels < 11.2 ng/ml (HR 0.15; 95% CI 0.03–0.58; *P* = 0.004) were found to be significantly favorable factors for OS (Table 2). Consequently, the median OS of patients with ≥ 26% lymphocytes or PSA levels < 11.2 ng/ml was significantly longer than that in their counterparts in the KRM-20 (median OS, not reached vs 16.4 months; *P* = 0.02 or median OS, not reached vs 16.3 months; *P* = 0.003, respectively; Fig. 3c, d).

**Table 2:** Cox proportional hazards regression analysis of associations between potential factors and overall survival in the 25 CRPC patients

Factors	Cut-offs^a^	Univariate			Multivariate		
		HR	95% CI	*P* value	HR	95% CI	*P* value
Lymphocytes, %	≥ 26 vs < 26	0.09	0.005–0.56	0.006	0.30	0.08–0.93	0.04
PSA, ng/ml	< 11.2 vs ≥ 11.2	0.22	0.06–0.60	0.003	0.15	0.03–0.58	0.004
Performance status	0 vs 1	0.36	0.09–2.45	0.25	0.51	0.15–2.32	0.34
Gleason score	< 8 vs ≥ 8	0.45	0.10–3.05	0.35	0.24	0.06–1.09	0.06
Neutrophils, %	< 65 vs ≥ 65	0.51	0.16–1.16	0.1	–	–	–
Treg, %	< 2.8 vs ≥ 2.8	0.63	0.21–1.75	0.38	–	–	–
MDSC, %	< 9.6 vs ≥ 9.6	0.72	0.25–2.02	0.52	–	–	–
Age, years	≥ 71 vs < 71	0.75	0.19–3.11	0.68	–	–	–
IgG, FIU	≥ 1142 vs < 1142	0.79	0.31–2.03	0.62	–	–	–
CTL, spots	≥ 4 vs < 4	1.04	0.41–2.82	0.94	–	–	–

Of the 25 patients, 9 died

CTL, cytotoxic T lymphocytes; FIU, fluorescence intensity units; HR, hazard ratio; IgG, immunoglobulin G; MDSC, myeloid-derived suppressor cells; Treg, regulatory T cells

^a^Cut-offs are based on median values

## Discussion

This randomized phase II trial of KRM-20 in combination with docetaxel and dexamethasone for patients with CRPC demonstrated a similar PSA decline, increased immune responses, and reduction in MDSC compared with docetaxel and dexamethasone treatment alone. PSA decline in several clinical trials for CRPC have been associated with longer survival [19]. However, improvements in OS in patients with CRPC may be observed without differences in PSA response rates [10], and PSA decline is not considered a true response. These results suggest the difficulty in clinical assessment of the phase II study setting for CRPC.

Active specific immunotherapy using either TAAs or their peptides has failed to provide clinical benefits for cancer patients in the large number of clinical trials since the 1990s, and the mechanisms involved in the failure have not been clarified [20–22]. Furthermore, this study did not demonstrate a longer OS or PFS than with the placebo. To understand the mechanisms involved in the failure of KRM-20, we predicted subsets of patients who will respond to KRM-20 with good clinical effects. Subgroup analysis suggested that this treatment is favorable for CRPC patients with ≥ 26% lymphocytes or PSA levels < 11.2 ng/ml. Although further validation is required, this finding is novel and helpful to prolong survival in patients with CRPC treated by peptide vaccinations.

In this study, assessment of HLA-matched peptide-specific IgG and CTL was conducted primarily to evaluate immune responses to the combination therapy with KRM-20. A significant increase in both HLA-matched peptide-specific IgG and CTL responses was observed in patients receiving the combination of KRM-20 with docetaxel and dexamethasone compared with patients in the placebo arm. The administration of cytotoxic chemotherapy agents, such as docetaxel, induces bone marrow suppression, and has been believed to negatively affect immune responses induced by the cancer vaccine. However, recent data suggest that many widely used chemotherapeutic agents have beneficial immunomodulatory effects thorough several mechanisms, including cytokine production, T cell infiltration of tumors, and maturation of dendritic cells [23–25]. The dose and scheduling of docetaxel in combination with cancer vaccines remain unknown. In a murine model, the combination of repeated melanoma tumor cell vaccinations with a more clinically relevant dose of docetaxel did not impede T-cell activation [26]. A previous randomized phase II trial of a cancer vaccine in combination with the clinical dose of docetaxel for metastatic CRPC patients did not inhibit vaccine-specific T cell reactivity [27, 28], and the present study demonstrated that KRM-20 in combination with docetaxel and dexamethasone increased HLA-matched peptide-specific IgG and CTL responses. Several clinical trials have suggested that cancer vaccines alter the clinical effects of subsequent docetaxel. An early phase trial of an adenovirus-based vaccine targeting p53 in patients with non-small cell lung cancer reported higher objective responses to salvage chemotherapy initiated after vaccine treatment [29]. Similarly, in a survival analysis of the pivotal randomized phase III IMPACT trial, patients who received sipuleucel-T prior to docetaxel demonstrated longer survival than patients receiving placebo prior to docetaxel (*P* = 0.03) [2]. However, although not exactly different from the current trial, two phase III trials (VITAL-1 and VITAL-2) of GVAX which was composed of two human prostate cell lines LNCaP and PC3 as antigen source, were failed to demonstrate survival benefit. VITAL-2 compared the combination of GVAX plus docetaxel with standard docetaxel and prednisone in men with symptomatic CRPC. The VITAL-2 study was terminated early due to increased deaths in the vaccine arm. The VITAL-1 was also terminated based on a result of less than a 30% chance of meeting an improved survival end point. Regarding the GVAX failure, it has been pointed out that the lack of placebo, docetaxel dose, and timing are not taken into account [30].

Another concern is the immune suppression caused by the continuous administration of low-dose dexamethasone with the peptide-based cancer vaccine. However, recent clinical trials found that combination therapy of low-dose dexamethasone with a peptide-based vaccine induced positive CTL responses, and a longer PSA PFS and OS than the peptide-based vaccine alone [31, 32].

In the current study, the number of MDSC, but not Treg, in patients in the KRM-20 arm decreased after treatment. Immune inhibition caused by MDSC in cancer patients has been reported in a number of studies. The increase in circulating MDSC also correlates with PSA levels and tumor burden in patients with CRPC [33]. Several preclinical studies reported that docetaxel combined with a cancer vaccine depleted circulating or tumor-infiltrating MDSC with CTL responses in murine tumor models by direct alteration of MDSC signaling, phenotype, and function [12, 13, 34]. These findings suggest potential clinical benefits by addition of docetaxel to the current immunotherapy.

The limitation of the present study was that the similar PSA decline, increase in immune responses, and decrease of MDSC in the KRM-20 arm did not lead to a longer PFS or OS than those in the placebo arm. This may have been due to the small number of patients and study design not comparing the difference in survival between the two arms. Further randomized clinical trials with a study design where the primary endpoint is the comparison of survival between KRM-20 in combination with or without docetaxel are needed.

The safety and tolerability were similar to the known profiles for docetaxel and peptide-based vaccines [10, 15]. The most common AEs were grade 1 or 2 injection site reactions in both arms. The main cause of grade 3 or higher AEs was hematology toxicity due to docetaxel, and these AEs developed in almost 40% of the patients in each arm. The addition of KRM-20 in combination with docetaxel and dexamethasone was feasible without increased toxicity.

In conclusion, KRM-20 in combination with docetaxel and dexamethasone for patients with CRPC resulted in a similar PSA decline, increased immune responses, and reduced MDSC compared with docetaxel and dexamethasone treatment alone. Subgroup analysis suggested that this treatment is favorable for CRPC patients with ≥ 26% lymphocytes or PSA levels < 11.2 ng/ml. Although patients in the KRM-20 arm had a slightly longer OS than those in the placebo arm, this study did not demonstrate any survival benefits. Further large-scale clinical trials comparing OS are required to confirm the clinical benefits of this treatment.

## Electronic supplementary material

Below is the link to the electronic supplementary material.

Supplementary file1 (PDF 256 kb)

## Abbreviations

CRPC: Castration-resistant prostate cancer
CTL: Cytotoxic T lymphocytes
ECOG: Eastern Cooperative Oncology Group
HLA: Human leukocyte antigen
IgG: Immunoglobulin G
MDSC: Myeloid-derived suppressor cells
OS: Overall survival
PBMC: Peripheral blood mononuclear cells
PD: Progressive disease
PFS: Progression-free survival
PSA: Prostate-specific antigen
TAA: Tumor-associated antigen
Treg: Regulatory T cells
