# Supplementary material for: Mixed 20-peptide cancer vaccine in combination with docetaxel and dexamethasone for castration-resistant prostate cancer: a randomized phase II trial
Source: Cancer Immunol Immunother. 2020 Feb 5;69(5):847–57. doi: 10.1007/s00262-020-02498-8 (PMC7183507; doi:10.1007/s00262-020-02498-8)
Supplement: Supplementary file 1 — Supplementary file1 (PDF 256 kb) [file 262_2020_2498_MOESM1_ESM.pdf]

Supplementary Table 1. Name, source TAA, position, amino acid sequence, and HLA type in the mixed 20-peptide vaccine (KRM-20)

Peptide name	Source TAA	Position of peptide	Amino acid sequence	HLA type
SART3-109	SART3	109-118	VYDYNCHVDL	A24, A3 family[a], A26
Lck-208	p56 lck	208-216	HYTNASDGL	A24
PAP-213	PAP	213-221	LYCESVHNF	A24
PSA-248	PSA	248-257	HYRKWIKDTI	A24
EGFR-800	EGF-R	800-809	DYVREHKDNI	A24
MRP3-1293	MRP3	1293-1302	NYSVRYRPGL	A24
Lck-486	p56 lck	486-494	TFDYLRSVL	A24
Lck-488	p56 lck	488-497	DYLRSVLEDF	A24
PSMA-624	PSMA	624-632	TYSVSFDSL	A24
PTHrP-102	PTHrP	102-111	RYLTQETNKV	A24
CypB-129	Cyclophilin B	129-138	KLKHYGPGWV	A2, A3 family[a]
Lck-246	p56 lck	246-254	KLVERLGAA	A2
WHSC2-103	WHSC2	103-111	ASLDSDPWV	A2, A3 family[a]
UBE-43	UBE2V	43-51	RLQEWCSVI	A2
WHSC2-141	WHSC2	141-149	ILGELREKV	A2
HNRPL-140	HNRPL	140-148	ALVEFEDVL	A2, A3 family[a], A26
SART3-302	SART3	302-310	LLQAEAPRL	A2
SART3-734	SART3	734-742	QIRPIFSNR	A3 family[a]
Lck-90	p56 lck	90-99	ILEQSGEWWK	A3 family[a]
Lck-449	p56 lck	449-458	VIQNLERGYR	A3 family[a]

[a] A3 family, HLA-A3, A11, A31, and A33

Abbreviations: CypB, cyclophilin B; EGFR, epidermal growth factor-receptor ; HLA, human leukocyte antigen; HNRPL, heterogeneous nuclear ribonucleoprotein L; Lck, $p56^{lck}$; MRP3, multidrug resistance-associated protein 3; PAP, prostatic acid phosphatase; PSA, prostate-specific antigen; PSMA, prostate-specific membrane antigen ; PTHrP, parathyroid hormone-related peptide ; SART3, squamous cell carcinoma antigens 3; TAA, tumor-associated antigen; UBE2V, ubiquitin-conjugated enzyme variant Kua; WHSC2, Wolf-Hirshhorn syndrome critical region 2.

Supplementary Table 2. HLA-matched peptide-specific IgG and CTL responses in the KRM-20 arm

Pts. No.	HLA type	No. of HLA-matched peptides	IgG response (No. of positive peptides)	CTL response (No. of positive peptides)
20201	A24/A11	16	negative (0)	positive (2)
20203	A24/A11	16	negative (0)	negative (0)
20206	A24/A33	16	negative (0)	positive (1)
20301	A33/A26	8	positive (8)	negative (0)
20302	A2/A24	17	negative (0)	negative (0)
20303	A2/A24	17	positive (4)	negative (0)
20305	A24	10	negative (0)	negative (0)
20401	A2/A26	9	negative (0)	negative (0)
20405	A24/A26	12	positive (1)	negative (0)
20406	A2/A24	17	positive (1)	negative (0)
20407	A24/A11	16	negative (0)	negative (0)
20502	A2/A24	17	positive (2)	negative (0)
20601	A11/A26	8	negative (0)	negative (0)
20602	A2/A33	13	negative (0)	negative (0)
20603	A24	10	positive (10)	positive (3)
20702	A2/A26	9	negative (0)	positive (1)
20703	A24/A31	16	negative (0)	negative (0)
20801	A24	10	negative (0)	negative (0)
20904	A24/A31	16	negative (0)	negative (0)
20906	A24/A11	16	negative (0)	negative (0)
21002	A2/A26	9	positive (2)	negative (0)
21005	A24/A11	16	negative (0)	negative (0)
21007	A2/A11	13	positive (4)	positive (2)

Abbreviations: CTL, cytotoxic T lymphocytes; HLA, human leukocyte antigen; IgG, immunoglobulin G

Supplementary Table 3. Summary of in-study adverse events

	KRM-20 arm (n = 23)					Placebo arm (n = 26)			
	Any grade	Grade 3	Grade 4	Grade 5		Any grade	Grade 3	Grade 4	Grade 5
Injection site reaction	16 (70)	0	0	0		10 (39)	0	0	0
Alopecia	14 (61)	0	0	0		17 (65)	0	0	0
Neutropenia	11 (48)	2 (9)	7 (30)	0		15 (58)	3 (12)	6 (23)	0
Peripheral neuropathy	11 (48)	0	0	0		13 (50)	0	0	0
Fatigue	9 (39)	0	0	0		2 (8)	0	0	0
Upper respiratory infection	7 (30)	0	0	0		1(4)	0	0	0
Leucopenia	5 (22)	3 (13)	1 (4)	0		1 (4)	1 (4)	0	0
Fever	5 (22)	1 (4)	0	0		3 (12)	0	0	0
Peripheral edema	5 (22)	0	0	0		2 (8)	0	0	0
Diarrhea	5 (22)	0	0	0		7 (27)	0	0	0
Appetite loss	5 (22)	1 (4)	0	0		4 (15)	0	0	0
Oral mucositis	4 (17)	0	0	0		5 (19)	0	0	0
Nail discoloration	4 (17)	0	0	0		0	0	0	0
Joint pain	3 (13)	0	0	0		4 (15)	0	0	0
Dry skin	3 (13)	0	0	0		1 (4)	0	0	0
Anemia	2 (9)	1 (4)	0	0		2 (8)	1 (4)	0	0
Decreased lymphocyte count	2 (9)	0	0	0		2 (8)	1 (4)	0	0
Febrile neutropenia	0	0	0	0		2 (8)	1 (4)	1 (4)	0
Pneumonia	0	0	0	0		3 (11)	0	0	2 (8)
Fracture	0	0	0	0		2 (8)	1 (4)	0	0
Decreased platelet count	0	0	0	0		1 (4)	1 (4)	0	0
Depression	0	0	0	0		1 (4)	1 (4)	0	0

Data are number of patients (%); patients may have had more than one event. All grade 1 or 2 adverse events developing in >10% of patients are reported. All grade 3, 4, and 5 adverse events are reported.

Protocol

Protocol: Phase 2, randomized, placebo-controlled study of docetaxel in combination with a mixed 20-peptide vaccine for patients with castration-resistant prostate cancer (UMIN000011028)

Background

The prognosis of patients with castration-resistant prostate cancer remains poor. Treatments that can provide stable disease control with long-term survival benefits are needed. One such treatment is considered to be the combination therapy of a cancer vaccine with chemotherapy. However, the optimal combination therapy using a cancer vaccine with docetaxel for chemotherapy-naïve patients with castration-resistant prostate cancer remains unknown.

Aims

We aim to examine whether a novel cancer vaccine consisting of 20 mixed peptides (KRM-20) designed to induce cytotoxic T lymphocytes in combination with docetaxel and dexamethasone enhances the anti-tumor effects in patients with castration-resistant prostate cancer.

Methods

Study design and population

This is a double-blind, placebo-controlled, randomized phase 2 study. Chemotherapy-naïve patients with progressive castration-resistant prostate cancer will be enrolled from 10 medical centers in Japan.

Inclusion criteria

The subjects must satisfy the following conditions.

1. Patients must be diagnosed as prostate cancer pathologically at the initial treatment.

2. Patients who had progressive disease after androgen deprivation therapy (ADT) either by surgical castration, gonadotropin-releasing hormone or antagonist treatment.

Progressive disease while receiving ADT, defined by any 1 of the following:

1) At least two consecutive rises in serum PSA obtained at a minimum of 1-week intervals.

2) Measurable disease with ≥50% increase in the sum of the cross products of all measurable lesions, or the development of new measurable lesions by RESIST.

3) Non-measurable (bone) disease consisting of new areas of uptake by bone scan consistent with metastatic disease compared to previous imaging.

3. Patients have serum PSA level ≥2 ng/mL

4. Anti-androgen therapy is discontinued for at least 4 weeks before the first vaccination for patients receiving flutamide and 6 weeks for those receiving bicalutamide.
5. Patients continue to stay on medical treatment such as LHRH agonists of LHRH antagonists to maintain testosterone level of 0.5ng/mL
6. Patients must be positive for HLA-A2, HLA-A24, HLA-A26 or HLA-A3 super type (A3, A11, A31, A33).
7. Written informed consent must be obtained from patients.
8. Patients must be more 20 year-old.
9. Patients must be at a score level
of 0-1 of performance status (ECOG).
10. Patients must be expected to survive more than 6 months.
11. Patients must satisfy the followings:
WBC ≥ 3,000/mm^3、 Neutrophil ≥ 2,000/mm^3, Lymphocyte ≥1,000/mm^3, Hb ≥ 8.0g/dl, Platelet ≥ 100,000/mm^3, Serum Creatinine ≤ 2 times upper limit of normal, Total Bilirubin ≤ 1.5 times upper limit of normal, AST, ALT ≤ 2 times upper limit of normal

Exclusion criteria
The following patients must be excluded:
1. Patients who had received chemotherapy using docetaxel any time before the treatment.
2. Patients who had received pre-therapies including chemotherapy or immunotherapy within 28 days before the treatment.
3. Patients who had received radiotherapy or strontium-89 within the last 8 weeks before the treatment.
4. Patients with severe symptoms (active and severe infectious disease, circulatory disease, respiratory disease, kidney disease, immunodeficiency, disturbance of coagulation).
5. Patients with active multiple cancers
6. Patients with the past history of severe allergic reactions.
7. Patients who do not agree with contraception during treatment and until 70 days after treatment.
8. Patients who had enrolled in another trial within 3 months or who are treating in another trial.
9. Patients who had received any peptides consist of a mixed 20 peptides (KRM-20).
10. Patients who are difficult to participate in this trial because of psychiatric symptoms.
11. Patients who are judged inappropriate for the clinical trial by doctors.

Randomization

After assessment of eligibility and appropriate consent, eligible patients are randomly assigned in a 1:1 ratio to receive either KRM-20 with docetaxel and dexamethasone (study arm) or placebo with docetaxel and dexamethasone (control arm) using a minimization technique with the following stratification factors: age (<65 or ≥65 years old) and PSA (<20 or ≥20 ng/ml) at the clinical research unit of Kurume University in Kurume, Japan.

Intervention

Patients will receive either KRM-20 (20 mg/0.5ml) or placebo (0.5ml) mixed with incomplete Freund's adjuvant (Montanide ISA-51VG; Seppic, Paris, France) subcutaneously on days 1, 8, 15, 22, and 29 with oral dexamethasone (1 mg) once daily on days 1 to 36. On day 36, one hour after receiving docetaxel at 70 mg/m^2 intravenously, patients will receive subcutaneous KRM-20 or placebo injection. Treatment with docetaxel and the study drug is repeated every 3 weeks for up to 5 cycles, and oral dexamethasone is continued once daily until the end of the study. Dosing delay and reduction for docetaxel is permitted if toxic effects are noted. Docetaxel may be held for less than 2 weeks until recovery and reduced to 60 or 50 mg/m^2 in the event of neutropenia (< $2,000/mm^3$), platelets < $100,000/mm^3$, hemoglobin < 8 g/dl, total bilirubin > 1·5 x ULM, transaminase > 2 x ULM, or serum creatinine > 2 x ULM. If docetaxel is held for more than 3 weeks, patients are removed from protocol treatment. After the study, patients who received protocol treatment will be followed up for 3 years for survival analyses.

Endpoints

The primary endpoint in this study was the comparison of each treatment arm for the rate of > 50% PSA decline from baseline. Based on the previous report, the assumed rate of > 50% PSA decline was 65% in the KRM-20 arm and 25% in the placebo arm. The target sample size was 50 assuming an ineligibility rate of approximately 10%. Sample size computation based on the large sample test was performed with the following assumptions: type I error rate = 0.05, power 80% and the ratio of the two groups as 1:1. Secondary endpoints are safety, immune responses, progression-free survival (PFS), and overall survival (OS). The Student’s t-test and chi-square test will be used to compare quantitative and categorical variables among safety profiles and immune responses to the treatment, respectively. PFS and OS data for each arm will be

analyzed using the Kaplan-Meier method. The log-rank test will be used to compare the survival curves, and Cox proportional hazard analysis to estimate hazard ratios (HR). The confidence intervals (CI) reported are 95%. Statistical analyses are performed using SAS software version 9·1 (SAS Institute, Cary, NC) with a two-sided significance level of 5%. All analyses will be on an intention-to-treat basis.

Funder

The Ministry of Health, Labour and Welfare of Japan (ID 2014110358 to M. Noguchi).

Date trial started

Jul 1, 2013

Expected date of completion

Jul, 2017
